# Micro-SORS-Based Protocol for the Noninvasive Depth-Resolved Study of Degraded ABS in Cultural Heritage

**DOI:** 10.1021/acs.analchem.6c00327

**Published:** 2026-07-14

**Authors:** Kevin Ambrogioni, Chiara Castiglioni, Francesca Rosi, Claudia Conti, Matteo Passoni, Pavel Matousek, Irene Bargagli, Alessandra Botteon

**Affiliations:** † Politecnico di Milano, Department of Energy, via Lambruschini 6, 20156 Milano, Italy; ‡ Politecnico di Milano, Department of Chemistry, Materials and Chemical Engineering “Giulio Natta”, piazza Leonardo da Vinci 32, 20131 Milano, Italy; § National Research Council, Istituto di Science e Tecnologie Chimiche “G. Natta” (CNR-SCITEC), Via Elce di Sotto 8, 06123 Perugia, Italy; ∥ National Research Council, Institute of Heritage Science (CNR ISPC), via Roberto Cozzi 53, 20125 Milano, Italy; ⊥ Central Laser Facility, Research Complex at Harwell, STFC Rutherford Appleton Laboratory UKRI, Oxford OX11 0QX, United Kingdom

## Abstract

In the field of cultural heritage, there is an urgent need to enhance our understanding of the composition of plastic materials and their degradation processes, especially given their susceptibility to relatively rapid deterioration. Among these materials, acrylonitrile–​butadiene–styrene (ABS), commonly found in museum collections, is particularly vulnerable to photoaging, primarily affecting the polybutadiene (PB) phase through oxidation and cross-linking, significantly compromising its mechanical properties. While noninvasive techniques have been successfully applied to characterize surfaces of ABS-based objects, a major limitation exists in terms of assessing the depth and spatial distribution of degradation without invasive sampling. This study introduces microspatially offset Raman spectroscopy (micro-SORS) as a noninvasive method to investigate artificially aged turbid ABS-based objects at both the surface and the subsurface level. First, micro-SORS spectra were collected using both a benchtop and an in-house-developed micro-SORS portable prototype, demonstrating the potential of the method for in situ diagnostics. Then, a simplified physical model based on light transport in turbid material is developed to describe the UV–vis photo-oxidation process. Lastly, a protocol is proposed for estimating the degradation depth in turbid ABS-based materials by interpreting micro-SORS measurements through the UV–vis degradation model. The model is initially calibrated through a few sacrificial samples to estimate the physical parameters. Then, the approach operates fully noninvasively, providing quantitative insights into subsurface aging phenomena. By providing a new strategy for depth-resolved aging assessment, this work bridges a key methodological gap in polymer conservation and paves the way for more informed treatment strategies and future noninvasive diagnostic applications.

## Graphical abstract

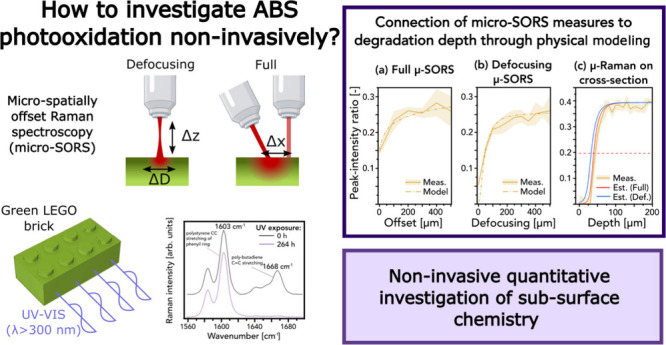

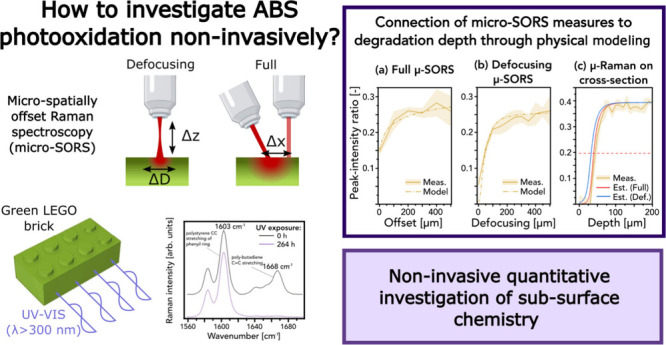

## Introduction

An ongoing challenge in the field of cultural heritage is the development of effective strategies for conserving plastic items, which are increasingly prevalent in contemporary art and design. Among these materials, the copolymer acrylonitrile–​butadiene–styrene (ABS) is one of the most commonly found plastics in museum collections.

Assessing the conservation state of ABS objects is essential for planning conservation actions. Indeed, ABS is particularly vulnerable to deterioration under exposure to light and elevated temperatures. Numerous studies have shown polybutadiene (PB), i.e., the rubbery component of the copolymer, to be the most susceptible to degradation, undergoing radical oxidation and subsequent cross-linking of the PB oxidized phase. Progressive aging may also trigger depolymerization of the styrene–acrylonitrile (SAN) fraction. The degradation process results in a modification of the mechanical properties of the material, including reduced impact resistance, reduced elasticity, and embrittlement.^1–8^

Given the unique constraints of working with irreplaceable cultural heritage artifacts, noninvasive analytical methodologies are critically important. The same requirement for the noninvasive assessment of materials underpins a wide range of disciplines, notably biomedical diagnostics and forensic investigations. In the field of cultural heritage, ABS-based materials are often turbid (nontransparent, highly diffusive), as they are typically mixed with colorants and opacifiers. This characteristic makes ABS-based materials more challenging to study at the subsurface level in a noninvasive way. To date, these materials have been studied by analytical techniques to monitor the chemical changes of ABS surfaces using vibrational spectroscopies^9–16^ (IR and Raman) and, recently, through correlative Brillouin and Raman microspectroscopy^8^ (BRaMS). Furthermore, the in-depth resolved relaxation behavior of ABS has been explored using relaxometry nuclear magnetic resonance spectroscopy.^8^ Some effort has also been made to analytically separate surface and subsurface chemistry by exploiting the different penetration depth of IR light in the near and mid-range.^8^ However, a significant gap still exists in the ability to noninvasively assess the depth and spatial distribution of degradation products within ABS-based artifacts. This shortcoming limits the accuracy of the conservation diagnostics and, consequently, constrains the development of optimum conservation treatments.

The present study proposes a noninvasive protocol, based on microspatially offset Raman spectroscopy (micro-SORS), to estimate the depth of photodegradation in artificially aged, turbid ABS-based samples, providing a quantitative assessment of their conservation state. Micro-SORS^17^ enables the retrieval of subsurface molecular information on turbid materials directly from the surface and has already been used to qualitatively characterize the diffusion and degradation of compounds in the subsurface of materials.^18,19^ In this context, micro-SORS offers a promising approach for characterizing degradation gradients in plastic materials, being both molecular and depth sensitive to the state of degradation. However, to go beyond a purely qualitative analysis and to be able to retrieve quantitative information about the degradation depth, physical models connecting the UV–vis-induced degradation to micro-SORS measurements are needed. In this study, we developed and validated a simplified analytical–physical model, based on the diffusion approximation for the UV–vis light transport equation.^20^ The model is initially calibrated on a limited number of cross sections of artificially aged ABS-based samples. Once this calibration phase is completed, the approach operates fully noninvasively, enabling the retrieval of the oxidized layer thickness from micro-SORS data obtained on turbid ABS-based materials with the same, and potentially similar, optical properties.

Micro-SORS experiments were performed using both a benchtop and an in-house-developed portable prototype; the latter was used to evaluate its potential for future in situ applications in real-case scenarios. This study fills a methodological gap in polymer degradation analysis by proposing a strategy to assess, through parameters determined quantitatively, depth-resolved aging in turbid ABS-based artifacts, paving the way for more informed conservation decisions and future noninvasive field diagnostics.

## Materials and Methods

### Samples

A set of light green LEGO bricks (The Lego Group) were studied as a reference material. In addition to ABS, the LEGO bricks contain phthalocyanine greens as pigments (namely Pigment Green 7 (PG7) and Pigment Green 36 (PG36)) and titanium dioxide (TiO_2_) as a pigment and/or opacifier.^4,8^

The set included a reference unaged brick and six artificially aged bricks. The aging was performed using a Cermax filtered xenon lamp (300 W, working range λ = 320–800 nm). The bricks were placed at a 50 cm distance from the light source. The samples were aged in a time span ranging from 12 to 264 h corresponding to the fluence values reported in Table 1.

**Table 1:** Label, Exposure Time, and Fluence

Label	Exposure time to UV–vis light (h)	Fluence (kJ/cm^2^)
UA	0	0
S1	12	7.7
S2	24	15.3
S3	48	30.7
S4	72	46.0
S5	120	76.7
S6	264	168.8

The samples were analyzed with micro-SORS, in both full and defocusing modalities, and conventional Raman microscopy on the cross sections (see details in the next section).

### Micro-SORS

#### Benchtop Prototype

Micro-SORS measurements were acquired with a Renishaw inVia Qontor micro-Raman prototype, equipped with a Peltier-cooled (−70 °C) NIR-enhanced CCD camera, a Leica DM2700 microscope, and a 785 nm excitation laser. The system was modified to enable micro-SORS measurements in a SORS full modality.^21^ An external laser probe was mounted on an SB 100 micrometric stage to set spatial offsets with respect to the Raman collection zone. The probe was equipped with an SLWD 20× objective (WD: 30.5 mm) to deliver the laser beam to the sample, bypassing the microscope internal optics, at approximately 38° with respect to the incidence plane (Figure 1). Due to this geometry, the spot of the laser possessed an elongated shape on sample surface, with a calculated major axis of 20 μm. For the collection of Raman photons, an LWD 20× objective (WD: 6.9 mm) was used. A laser power of 70 mW and an acquisition time of 10 s with 30 coadditions were used (300 s total acquisition time). The spectra were collected at a 0 μm offset and by progressively increasing the offset between the laser and collection points, up to 800 μm, with a 100 μm step. Three full micro-SORS sequences per sample were acquired. The defocusing micro-SORS^22^ measurements were acquired using a 50× microscope objective, a laser power of 35 mW, and an acquisition time of 6 s with 20 coadditions. The spectra were collected at the imaged (in focus) position and by progressively increasing the distance between the sample surface and the objective up to 500 μm distance, with a step size of 50 μm. Five defocusing micro-SORS sequences per sample were acquired.

**Figure 1:**
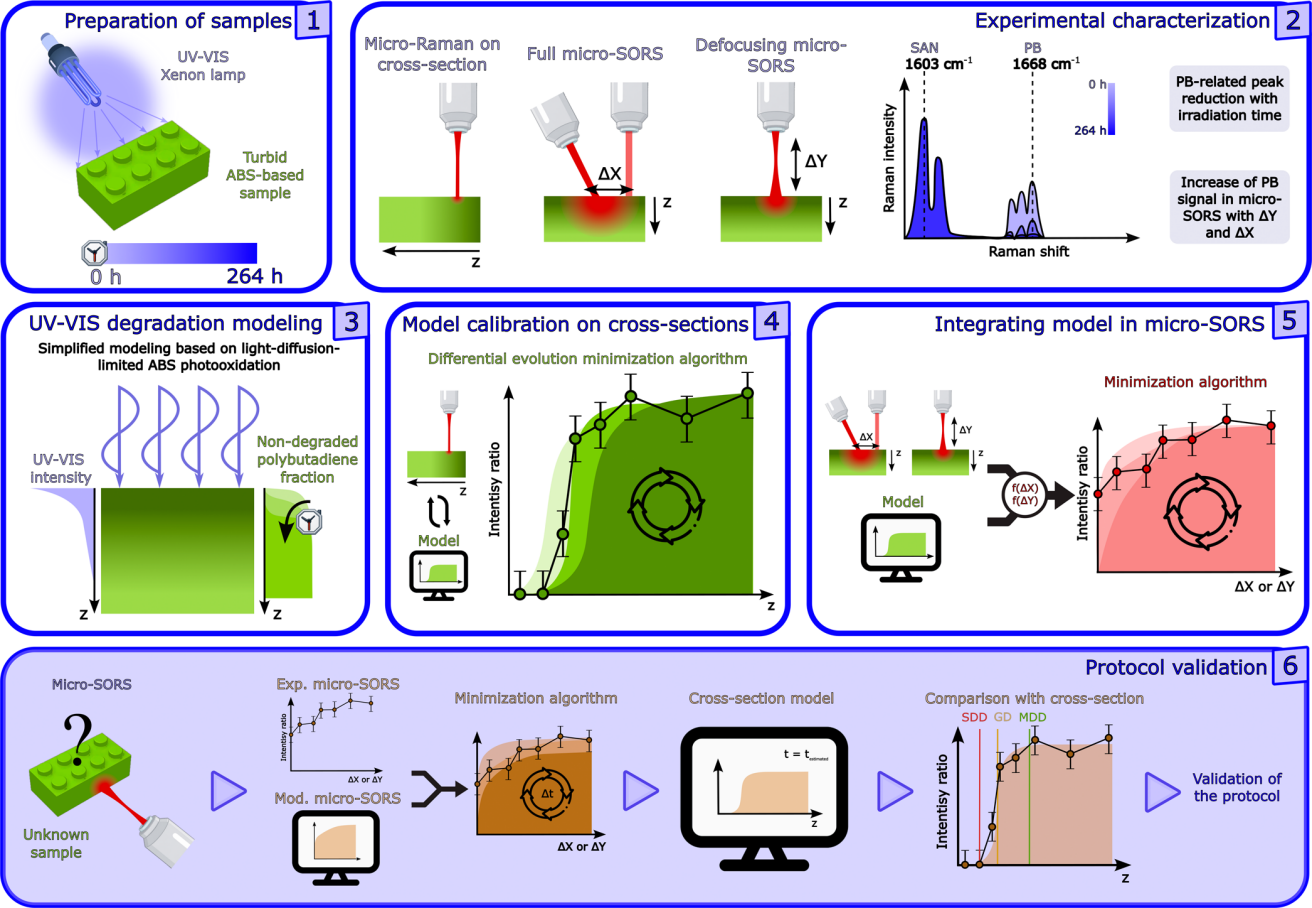
Graphical representation of the workflow used in this article.

#### Portable Prototype

The in-house portable prototype with a 785 nm excitation laser, described by Lux et al.,^23^ was used to collect the micro-SORS spectra. This instrument was implemented with a custom fiber bundle (Armadillo SIA – LV) used to project the offset spectra on the *y*-axis of the CCD, enabling the simultaneous acquisition of multiple spatially offset spectra without moving components, ranging from 0 to 545 μm. We acquired seven micro-SORS sequences per sample, using a laser power of 4.8 mW at the sample and an acquisition time of 50 s with 5 coadditions (250 s total acquisition time).

### Conventional Raman spectroscopy

The cross sections of samples S1, S4, S5, and S6 were obtained by breaking the bricks after immersing them in liquid nitrogen to reach the ductile-to-brittle transition temperature for the material, thus achieving a brittle rupture. The conventional Raman spectra were collected with the benchtop micro-Raman spectrometer described in the Benchtop Prototype section, from the surface to a 750 μm depth. A 10 μm step size was used from the surface to a 250 μm depth, while a 50 μm step size was used from 250 to 750 μm. The measurements were carried out in three different locations on each sample, using a 100× microscope objective, a laser power of 5 mW, and an acquisition time of 5 s with 5 coadditions (25 s total acquisition time).

### Raman Band Intensity Ratio Determination

The Raman spectrum of ABS shows strong bands assigned to vibrational modes of the different copolymer blocks. These bands enable monitoring the relative amounts of polystyrene, polybutadiene, and polyacrylonitrile monomers in the material. The strong, structured Raman band in the region 1630–1680 cm^–1^ is ascribed to C=C stretching normal modes localized on the polybutadiene segments. The C=C stretching vibrational frequency is sensitive to the configuration of the chemical units and gives rise to three main components assigned to the trans (1668 cm^–1^) and cis (1653 cm^–1^) configurations of 1,4-polybutadiene units and to the vinyl group (1640 cm^–1^) of the 1,2-polybutadiene units.^4,8,24^ The Raman spectra of the studied ABS samples clearly show the presence of the three C=C stretching components, suggesting that the polybutadiene sequences are characterized by configurational disorder. The very strong CC stretching Raman band observed at 1603 cm^–1^ is a marker of the aromatic rings belonging to the polystyrene segments.^4,8,24^ A common approach in micro-SORS analysis involves calculating the intensity ratio of selected Raman bands to track their variation with increasing spatial offset or defocusing, thereby monitoring changes in chemical composition as the probed depth increases. The intensity ratio between a Raman marker band of the polybutadiene units and a marker band of polystyrene serves as a proxy of the relative abundance of the two different chemical species in the material.

Assuming UV–vis irradiation preferentially modifies (i.e., degrades) polybutadiene units, the ratio between the peak intensity of the Raman band at 1668 cm^–1^ (C=C trans stretching of polybutadiene, *I*_1668 *cm* ^–1^_)^4,24^ and that of the band at 1603 cm^–1^ (CC stretching mode of the benzene ring, *I*_1603 *cm* ^–1^_)^4,24^ can be used to monitor the degradation affecting the polybutadiene component.

Our approach requires that one of the three individual C=C stretching band components, namely the 1668 cm^–1^ peak, is selected as representative of the polybutadiene phase. The choice is supported by three observations: (i) The 1668 cm^–1^ peak is the strongest one, thus guaranteeing the most reliable intensity estimate. (ii) The relative intensities of the three components of the C=C stretching band are almost stable in the whole set of measurements (see Figure S1 in the Supporting Information). (iii) A preliminary micro-SORS analysis based on band integral ratios showed very similar trends to those obtained when focusing on peak intensities (see Figure S2 in the Supporting Information). Although the Raman band integral is directly proportional to the probability of Raman transition, we used the band intensity (i.e., peak height), which handles weak and noisy signals more effectively. Observations (ii) and (iii) confirm that we can quantify the relative abundance of the polybutadiene phase through the number density of trans C=C bonds, which contribute to the Raman signal at 1668 cm^–1^. Due to the high amount of data to analyze, we developed a Python-based code to automatize the fitting procedure of the Raman spectra. To avoid effects related to baseline fluctuations, the “statistics-sensitive nonlinear iterative peak clipping” algorithm was chosen for performing the baseline correction, and an inspection in the Raman spectra from degraded samples was performed before starting the fitting procedure to guarantee that no degradation products were visible in the region of interest (see the Supporting Information for details on the automatized fitting procedure).

## Results and Discussion

In this section, the results are presented according to the following workflow. First, we introduce the micro-SORS measurements obtained from the artificially aged ABS samples, along with the Raman analyses of their cross sections. We then present the physical model we propose to describe the evolution of degradation in generic turbid ABS-based objects under increasing UV–vis irradiation doses. Lastly, we outline a protocol for the noninvasive quantitative characterization of the artificially aged ABS samples, developed by calibrating and validating the model using the experimental data. A scheme summarizing the workflow used in this article is presented in Figure 1.

### Micro-SORS and Cross-Section Measurements of UV–Vis-Aged Turbid ABS Samples

In Figure 2a, a comparison between the Raman spectra acquired on the surface of the UA and S6 samples is shown. The UA spectrum in Figure 2b shows the Raman bands at 1640, 1653, and 1668 cm^–1^ that are ascribed to the C=C stretching of the polybutadiene units in different configurations.^24^ These are almost indiscernible from the background in the aged sample S6. This phenomenon is attributed to the consumption of PB C=C bonds through UV–vis-light-induced oxidation.^8^ In the micro-SORS spectra, the intensity of C=C Raman bands progressively increases with increasing probed depth. This behavior highlights that the degradation of the material predominately affects its surface (see Figure 3), as expected for the process taken into consideration.

**Figure 2:**
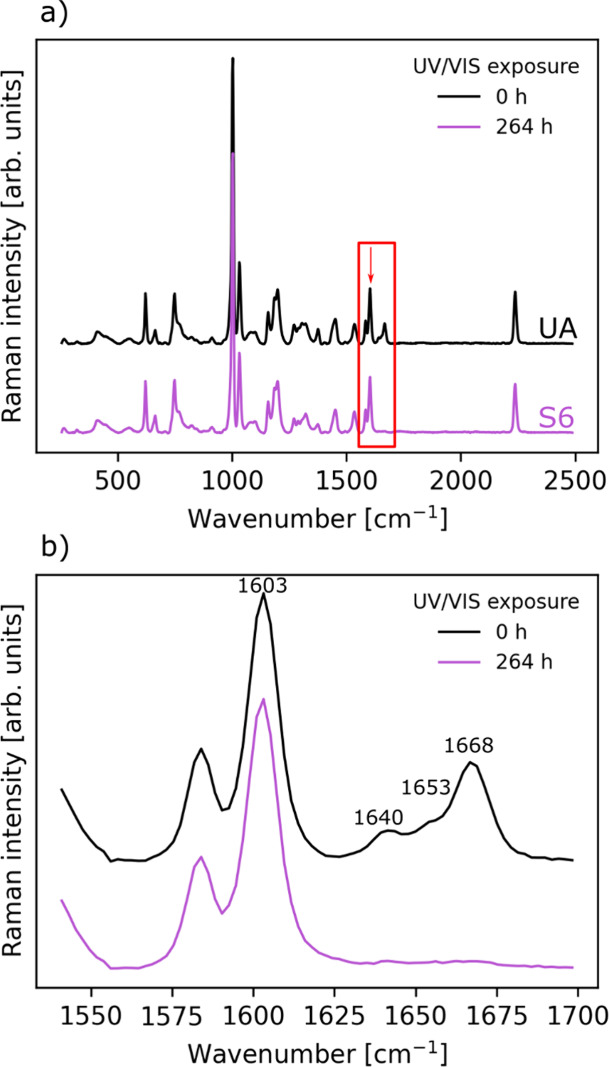
a) Raman spectra of the surface of samples UA and S6 (264 h), normalized to the intensity of the 1603 cm^–1^ Raman band (red arrow), ascribed to the CC stretching vibration mode of the benzene ring.^23^ The spectra are displaced for clarity. The red box frames the Raman bands shown in b). b) Zoomed-in section of the Raman spectra of the surface of samples UA and S6 (264 h). In the S6 spectrum, the Raman bands ascribed to polybutadiene (1640, 1653, and 1668 cm^–1^) are not visible.

**Figure 3:**
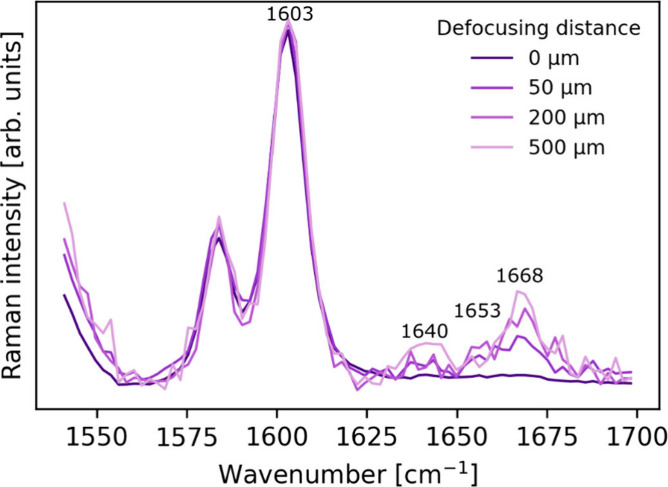
Defocusing micro-SORS sequence collected on S6 (264 h). The spectra are normalized to the 1603 cm^–1^ Raman band. Butadiene Raman bands become progressively more intense with increasing defocusing distance (namely 50, 200, and 500 μm).

As described in the Raman Band Intensity Ratio Determination section of the Materials and Methods, the bands at 1668 and 1603 cm^–1^ were deemed the most suitable for monitoring material degradation. In Figure 4, the ratios between the selected Raman band intensities obtained for defocusing micro-SORS (Figure 4a) and full micro-SORS measurements (Figure 4b) are reported. The ratios at the imaged position (Figure 4a) and at the 0 μm offset (Figure 4b) of all the samples reveal a clear anticorrelation with photodegradation time: the longer the exposure to light, the lower the ratio. This trend confirms the progressive depletion of PB C=C bonds at the surface.

**Figure 4:**
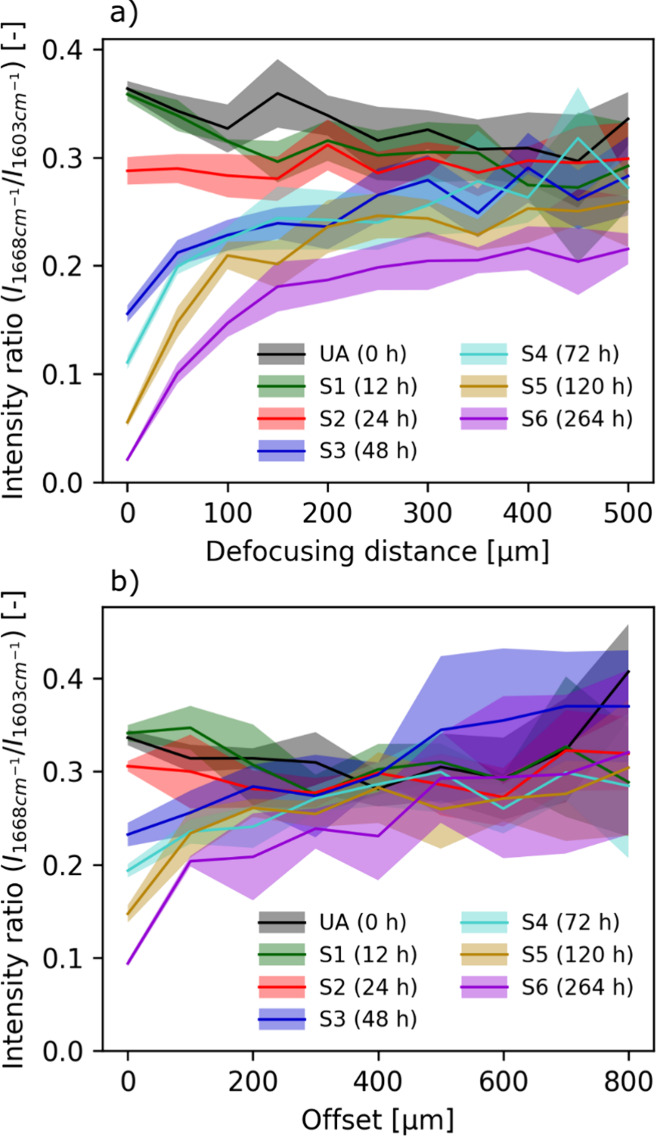
a) Defocusing and b) full micro-SORS ratio plots, obtained with spectra collected with the benchtop instrument.

The defocusing modality exhibits a higher lateral resolution at the imaged position and at small defocusing distances, compared to the full micro-SORS counterparts. This is ascribed to the use of a 50× microscope objective for defocusing, as opposed to the 20× objective used for full micro-SORS measurements. The use of the 20× objective for full micro-SORS is mandatory in this case due to the optical footprint of the benchtop prototype. It should be noted that both full and defocusing micro-SORS suffer from signal overlap between the unaged sample (UA) and the less decayed one (S1, 12 h), indicating that even the 50× magnification used for defocusing is insufficient to fully resolve them. At large defocusing distances, not all sample trends converge, e.g., S6 (264 h) remains distinct from the other samples, highlighting the surface effect of the defocusing approach.^25^ In contrast, full micro-SORS provides a better separation between surface and subsurface signals. All full micro-SORS measurements eventually reach a plateau at comparable ratio values, suggesting that the inner bulk of the ABS bricks remains unaltered. Using the portable instrument, some sample trends exhibit partial overlap (see Figure 5a–c). Nonetheless, the plots demonstrate the potential to distinguish the unaged sample (UA), an “intermediate” aged sample (S4, 72 h) and the most degraded one (S6, 264 h) (Figure 5d). Notably, the trends acquired with the portable device do not replicate the unequivocal monotonic increase in ratio values observed with the benchtop instrument, even in severely-degraded samples.

**Figure 5:**
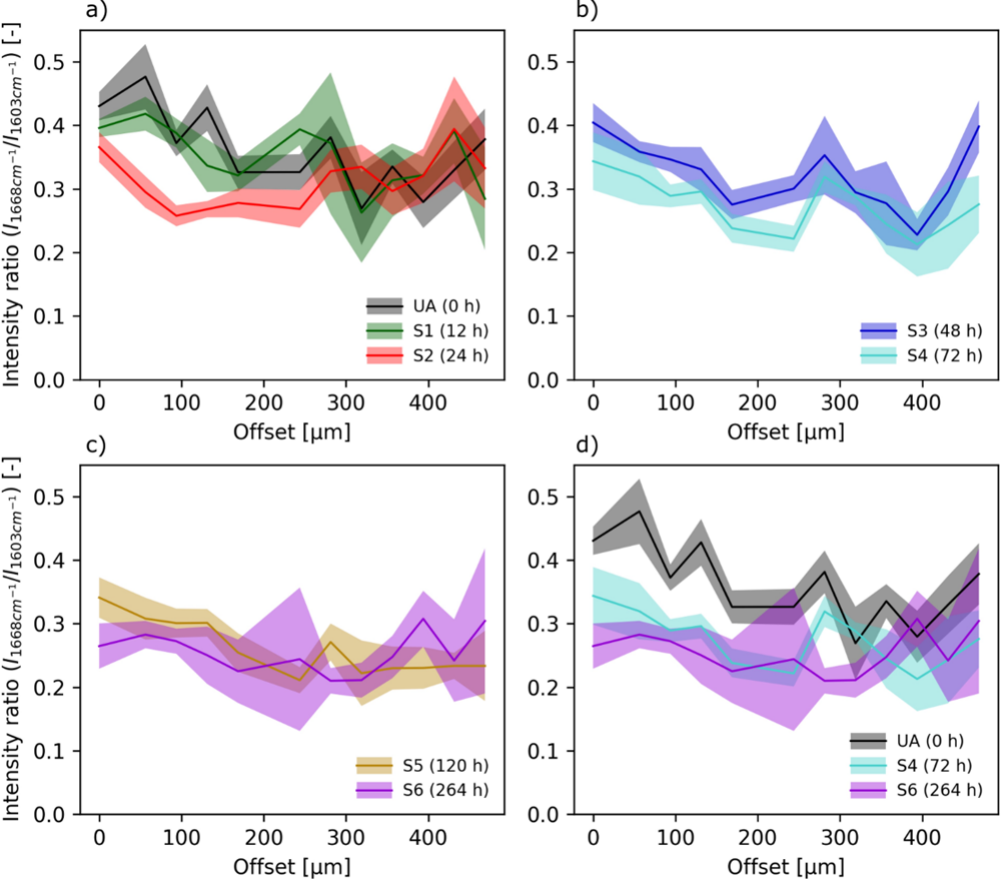
Full micro-SORS ratio plots obtained with the portable micro-SORS prototype. Samples are displayed in separate panels a), b) and c) to improve readability. In d), samples UA, S4 and S6 are compared.

This disagreement is not due to efficiency variations of the photon transport of the light through the fiber bundle of the portable setup affecting Raman intensity ratios (see Figure S3 in the Fiber Bundle Efficiency Test section in the Supporting Information). The most plausible causes of this discrepancy include the lower signal-to-noise ratio and greater spectral distortion observed in the portable system (see the Supporting Information). Additionally, the use of lower magnification objectives (10×) and suboptimal alignment between excitation and collection fibers likely reduced the lateral resolution and depth sensitivity. Nevertheless, being able to discern the state of degradation of materials, the results suggest that the portable micro-SORS prototype holds promise for the noninvasive in situ investigation of degraded ABS in cultural heritage objects.

Cross sections of samples S1, S4, S5, and S6 show results that are in line with the micro-SORS findings (see Figure 6): S1 does not show significant decay, and in S4 the thickness of the decayed material is shallower compared to that in S5 and S6.

**Figure 6:**
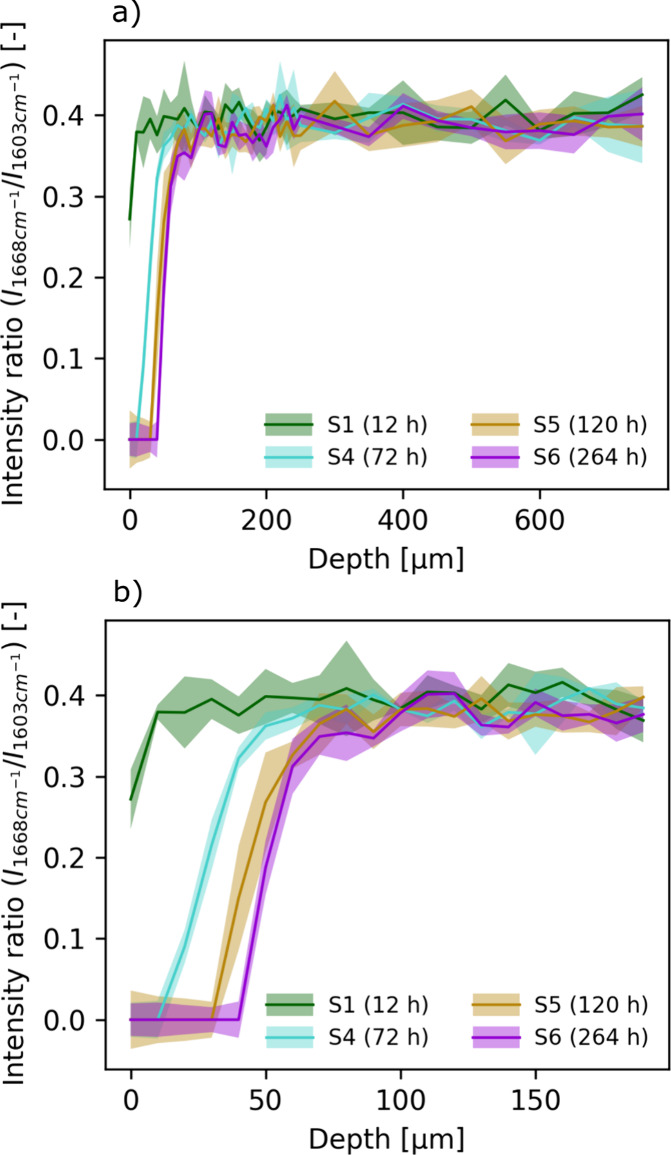
Ratio plots of the cross sections in the depth ranges of a) 0–750 μm and b) 0–200 μm.

To derive quantitative parameters for evaluating the degradation state of the material, specific depth values were selected at points exhibiting characteristic features of the degradation profile. These values effectively capture the progression of material degradation with increasing irradiation time:

- The depth at which the Raman signal of the 1668 cm^–1^ peak is not discernible from the spectral noise, i.e., the ratio is considered null. This value is representative of the portion of the material that can be considered almost completely degraded, and we call it the strong degradation depth (SDD).
- The depth at which the ratio between the 1668 and 1603 cm^–1^ peak intensities reaches the half-value between its minimum and its maximum in the degradation profile. This value is representative of the steepness of the curves. We call this value the gradient depth (GD).
- The depth at which the ratio between the 1668 and 1603 cm^–1^ peak intensities reaches the nondegraded value within its statistical uncertainty for the first time, i.e., the ratio is higher than 0.381. This value is representative of the depth at which the experimental observation cannot distinguish between the degraded and nondegraded material. It reasonably describes the maximum degradation depth (MDD).

The values of SDD, GD, and MDD for the analyzed samples are summarized in Table 2. Considering S1, the material has no sign of strong degradation. In fact, even on the surface, the 1668 cm^–1^ peak could be discerned from the background. The strong degradation region is first observed in S4 among the analyzed samples.

**Table 2:** Summary of the Degradation Depth Values Inferred by the Cross-Section Measurements

Sample	SDD (μm)	GD (μm)	MDD (μm)
S1	-	5.7 ± 1.5	30.0 ± 5.0
S4	10.0 ± 5.0	28.6 ± 2.6	70.0 ± 5.0
S5	30.0 ± 5.0	44.0 ± 6.8	80.0 ± 5.0
S6	40.0 ± 5.0	50.8 ± 2.7	100.0 ± 5.0

### Physical Model for the Propagation of Photoinduced Degradation in Turbid ABS-Based Objects

Under UV–vis irradiation (λ = 320–800 nm), ABS undergoes photo-oxidation in the butadiene phase, where reactive oxygen species progressively oxidize the double bonds, leading to chain scission, cross-linking, and the formation of oxidized functional groups.^26^ To enable a quantitative analysis of turbid ABS-based objects (i.e., ABS containing opacifiers and pigments) typical of contemporary art and design, a dedicated model is required to relate the state of conservation—specifically, the depth of the degraded material—to the micro-SORS measurements. To develop the model, the ratio between the Raman intensities of the C=C trans stretching of polybutadiene and the CC stretching mode of the benzene ring was considered as the probe (as presented in the Raman Band Intensity Ratio Determination section of the Materials and Methods) for the state of degradation.

The photodegradation reaction in ABS is mediated by the concurrent presence of (i) photons able to generate radicals, (ii) oxygen that permeates the material from the material–air surface, and possibly (iii) impurities or structural defects that facilitate radical formation and promote the initiation of the photo-oxidation process. Pigments and opacifiers could also participate in this complex reaction, which is at the base of the ABS photo-oxidation process happening in the UV–vis range and which leads to the modification of the C=C bonds of the butadiene unit. In a regime in which the radical reaction is not oxygen-diffusion-limited^27^ (i.e., the oxygen content is largely in excess with respect to the radical production induced by the photons, and the energy and the photon penetration are the driving factors), the modeling of the UV–vis photoinduced degradation should be based on the photon propagation through the material. This light-penetration-limited regime can be considered valid upon the satisfaction of two main hypotheses: the first considers the length scale of the degradation, i.e., the model is valid up to where oxygen is efficiently permeating in the material (around 100 μm in ABS),^26^ which is thereby consistent with the experimental data presented in the previous section; the second concerns the decay length of radiation within the material. The material must behave as a turbid medium for the incident radiation so that the photon density varies significantly within a thin region beneath the sample surface. While previous experimental data show pure ABS to be almost transparent to the considered light wavelength^28^ (i.e., λ = 320–800 nm)—and thus, the reaction is controlled by the oxygen diffusion layer—in the case under study and that commonly observed in cultural heritage, opacifiers and pigments increase the light scattering and the absorption of the composite material, confining the radiation penetration to a lower length scale. This consideration allows one to consider the UV–vis light transport in the material the limiting factor in the degradation process, thus increasing the typical time scale to reach the maximum degradation allowed by oxygen penetration. Under these main hypotheses, the following assumptions were made to develop a simplified physical model for describing the degradation process:

(i) The material is considered as a semi-infinite slab, consistent with the usual thickness of ABS plastic layers in art and design objects which is orders of magnitude greater than the penetration depth of UV–vis light in the material, and the diffusion approximation to light transport equation is supposed to hold.
(ii) The UV–vis illumination is planar and uniform. The scattering and absorption coefficients are considered quasi-constant in the most effective spectral region (i.e., 320 < λ < 450 nm, the most energetic component of the spectrum) for the oxidation process, i.e., the 1-group approximation^29^ to transport equation holds for the most degradation-effective components of the light. The polybutadiene and the polystyrene copolymer units are treated as two different weakly interacting chemical species, and the Raman bands assigned to the phenyl ring stretching and to the C=C stretching of the trans polybutadiene units are selected as marker bands to quantify the relative amount of the two chemical species.
(iii) The degradation of the phenyl rings, i.e., of the styrene component, is negligible with respect to that of the butadiene units, consistent with previous literature results, which show the latter to be more prone to degradation upon UV–vis-induced photo-oxidation.^4,24,26^

iv. The absorption coefficient of the UV–vis light in the degradation-effective spectral region is assumed to be constant over time, except for a contribution that depends on the material degradation state. Since we chose the amount of polybutadiene units (C=C bonds) as an indicator of the material’s degradation, the absorption coefficient variation with degradation is linearly related to the content of nondegraded polybutadiene. Conversely, the scattering coefficient of the material is assumed to remain constant during irradiation.

The description of light transport in the material is based on the diffusion approximation to light transport equation in absorbing and scattering materials.^20^ Under these assumptions, the light intensity (*I*) in the material, as a function of the depth from its surface (*z*), can be approximately described as follows:

$$I\left(z\right)\approx {\left(1-R\right)I}_{0}\;\exp\left(-\sqrt{3\left(\mu_{a}^{2}+\mu_{a}\mu_{s}\right)}z\right)$$ 1

where *I*_0_ is the UV–vis light intensity incident at the surface, *R* is the reflected light fraction, μ_*s*_ is the scattering coefficient of the material, and μ_*a*_ is the absorption coefficient of the material. The absorption coefficient of the material, under the fourth hypothesis, can be approximately modeled as μ_*a*_ = μ_*a*,0_ + *k*_*a*_ *n*_*C=C*_, where μ_*a*,0_ is the absorption coefficient in the degradation-effective spectral range but for a contribution that depends on the amount of polybutadiene units, i.e., the degradation state. This contribution accounts for changes of the bulk absorption coefficient due to the occurrence of the degradation process, which is quantified by the resulting number density of polybutadiene units, i.e., by the number density of C=C bonds in the material, *n*_*C=C*_. *k*_*a*_ is the linear parameter that relates the absorption coefficient to the nondegraded polybutadiene unit content.

Considering a linear relation between the UV–vis light intensity and the depletion of C=C bonds (i.e., a first order approximation of the photo-oxidation process) and considering eq 1, the spatial-temporal evolution of the degradation is described through a decay-like equation:

$$\frac{dn_{\mathrm{C=C}}}{dt}={-\sigma }_{d}{\left(1-R\right)I}_{0}\times \exp\left(-\sqrt{3\left(\mu_{a,0}+k_{a}n_{\mathrm{C=C}}\right)\left(\mu_{a,0}+k_{a}n_{\mathrm{C=C}}+\mu_{s}\right)}z\right)n_{\mathrm{C=C}}$$ 2

where *t* is the time from the start of the degradation process and σ_*d*_ is the average degradation cross section of C=C bonds in the considered UV–vis range for the photo-oxidation process in the presence of infinite oxygen content. The equation can be analytically solved only under the hypothesis of not-strongly-degraded regions or for early stages of degradation, i.e., when μ_*a*_ = μ_*a*,0_ + *k*_*a*_ *n*_*C=C*_ can be considered a constant. Otherwise, a numerical solution to the equation is needed. In the limit of not-strongly-degraded material (or the early stage of the degradation solution), the provided analytical solution is as follows:

$$n_{\mathrm{C=C}}\left(t,z\right)=n_{0,\mathrm{C=C}}\;\exp\left(-\sigma_{d}\left(1-R\right)I_{0}t\;\exp\left(-\sqrt{3\left(\mu_{a}^{2}+\mu_{a}\mu_{s}\right)}z\right)\right)$$ 3

where *n*_0,*C=C*_ is the number density of C=C bonds in the nondegraded material. It is then straightforward to relate the UV–vis degradation model to the Raman peak intensities. Considering, under the second hypothesis, a linear relation between the number density of nondegraded polybutadiene units and the intensity of the peak related to C=C trans stretching vibration and considering, because of the third hypothesis, the phenyl ring not to be influenced by the degradation process, one can relate the ratio (*r*(*t*, *z*)) between the Raman intensities of the C=C trans stretching vibration of polybutadiene and the CC stretching vibration mode of the phenyl ring with the degradation model through the estimation of the local number density of the undegraded C=C trans bonds in the material (*n*_*C=C*_(*t*, *z*)) computed through eq 2:

4 $$r\left(t,z\right)=\frac{I_{1668\;{\mathrm{cm}}^{-1}}}{I_{1603\;{\mathrm{cm}}^{-1}}}=\frac{\sigma_{\mathrm{Raman},\mathrm{C=C}}n_{\mathrm{C=C}}\left(t,z\right)}{\sigma_{Raman,ring}n_{ring}}$$

where σ_*Raman*,*C=C*_ is the Raman cross section for the C=C trans stretching vibration of polybutadiene, σ_*Raman*,*ring*_ is the Raman cross section for the CC stretching vibration mode of the phenyl ring, and *n*_*ring*_ is the number density of phenyl rings in the material, i.e., related to the number density of the styrene units in the material. Using eq 4 and the results of eq 2 (or eq 3 in the limit of not-strongly-degraded material), one can now relate the ratio between Raman intensities of the C=C trans stretching of polybutadiene and the CC stretching mode of the benzene ring to a local probe of the material state of degradation in depth. This local probe can then be used (see next section) to quantify the degradation depth from noninvasive micro-SORS measurements using an appropriate modeling of the Raman photon collection based on work by Castiglioni et al.^25^ It is, however, important to highlight that the hypotheses made to develop the simplified model pose limitations in its range of validity. First, it can be applied only to highly scattering ABS formulations, i.e., those containing opacifiers and pigments. Second, it can describe only the first steps of the material degradation, i.e., when the oxygen diffusion inside the material is efficient. In fact, when the material can be considered transparent to the UV–vis light or the degradation depth reaches a point at which oxygen diffusion is no longer efficient, the reaction becomes oxygen-diffusion-limited. In this case, a proper description should then account for the diffusion of the oxygen throughout the material depth in a reactive medium. In these conditions, while the retrieved degradation profile might have a similar shape to that of the light-propagation-limited regime, the time evolution of the degradation should be properly modeled based on the physics of oxygen diffusion in an oxygen reactive medium.

### Estimation of UV–Vis-Induced Degradation Depth in UV–Vis-Aged Turbid ABS Samples

To develop a strategy to assess and quantify the degradation state of turbid ABS material through noninvasive micro-SORS measurements, we developed a method based on the model presented in the previous section. First, we calibrated the model on a subset of samples’ cross sections (S1, S4, and S6) to define the parameters that describe the process in the studied material through the model. The data of the Raman spectroscopy on cross sections were considered as a local probe allowing us to directly fit the free parameters of eq 4 from experimental data. Then, we developed a model for the full and defocusing micro-SORS modalities based on work by Castiglioni et al.^25^ This model was fitted against the micro-SORS experimental data of S1, S4, and S6 to create a connection between the cross-section data (described via eq 4) and the micro-SORS measurements.

Given the data from the fitting procedures, we tested the strategy on one of the samples that were not used for the fitting procedure. The full and defocusing micro-SORS data were used as inputs to another fitting procedure, which returns the irradiation time (linearly related to the UV–vis fluence) and is able to minimize the error between the experimental micro-SORS data and the model (i.e., the connection between the degradation and the micro-SORS models). Finally, the irradiation time is used to estimate the degradation profile, and the latter is compared to experimental data through the parameters defined in previous sections. The results of the fitting procedures and of the proof-of-principle of the strategy application are presented in the following subsections.

#### Calibration of the Physical Model

The results from the cross sections and micro-SORS measurements were used to fit the parameters of the model introduced in the previous section. The ratios between the C=C trans stretching vibration of PB and polystyrene peaks of the S1, S4, and S6 sample cross sections, shown in Figure 6, were used as input data for a fitting procedure based on the differential evolution (DE) algorithm. The DE algorithm was chosen considering the strong nonlinearities of the model; the choice of the three samples for the calibration of the model was made to consider a slightly-degraded (S1), an intermediately-degraded (S4), and a strongly-degraded sample (S6). Four free parameters are used for the fitting (see below). These parameters heavily depend on the material composition; thus, they cannot be used for materials that are substantially different from the one under analysis. The fitting procedure was applied under two hypotheses, based on the considerations on the UV–vis light absorption coefficient:

(i) Not-strongly-degraded ABS, i.e., constant absorption coefficient.
(ii) Strongly-degraded ABS, i.e., varying absorption coefficient with degradation.

In the first case, the analytical approximation of eq 3 was used as a guess function for *n*_*C=C*_(*t*, *z*) in eq 4:

5 $$r\left(t,z\right)=C\;\exp\left(-Bt\;\exp\left(-\mu_{0}z\right)\right)$$

where *r*(*t*, *z*) is the quantity defined in eq 4. *C*, *B*, and μ_0_ were considered as free parameters of the fit. In this context, *C* = $\frac{\sigma_{Raman,\mathrm{C=C}}n_{\mathrm{C=C},0}}{\sigma_{Raman,ring}n_{ring}}$ represents the Raman intensity ratio between the two peaks for the nondegraded material, *B* = σ_*d*_(1 – *R*)*I*_0_ the degradation cross section multiplied by the radiation intensity incoming in the material, and μ_0_ = $\sqrt{3\left(\mu_{a}^{2}+\mu_{a}\mu_{s}\right)}$ the mean extinction coefficient of the UV–vis radiation in the diffusion approximation. The fit results are reported in Table 3.

**Table 3:** Summary of the Fitting Parameters Using the Not-Strongly-Degraded ABS Model

*C* (−)	*B* (1/h)	μ_0_ (1/μm)
0.396	4.99 × 10^–2^	5.83 × 10^–2^

In contrast, in the second case, the numerical integration of eq 2 was performed at each iteration of the fit procedure using an implicit Runge–Kutta method of the Radau IIA family of the fifth order.^30^ To keep the fitted function values in the range of [0, 1] and thus increase the fit stability, the following normalization was introduced for eq 2:

$$\{\begin{array}{l} \frac{d{ñ}_{\mathrm{C=C}}}{dt}=B\left(\exp\left(-\sqrt{3\left(\mu_{a,0}+K_{a}{ñ}_{\mathrm{C=C}}\right)\left(\mu_{a,0}+K_{a}{ñ}_{\mathrm{C=C}}+\mu_{s}\right)}z\right)\right){ñ}_{\mathrm{C=C}}\\ r=C{ñ}_{\mathrm{C=C}} \end{array}$$ 6

where *ñ*_*C=C*_ is the number density of C=C bonds normalized to the nondegraded value *ñ*_*C=C*_ = $\frac{n_{\mathrm{C=C}}\left(t,z\right)}{n_{\mathrm{C=C},0}}$ , *n*_*C=C*,0_ is the number of the C=C bonds in the nondegraded material, and *K*_*a*_ = *k*_*a*_ *n*_*C=C*,0_ is a linear parameter that relates the absorption coefficient to the polybutadiene unit content multiplied by the normalization factor. *C*, *B*, μ_0_, and σ_0,*a*_ were left as free parameters for the fit. Here, *C* = $\frac{\sigma_{Raman,\mathrm{C=C}}n_{\mathrm{C=C},0}}{\sigma_{Raman,ring}n_{ring}}$ represents the Raman intensity ratio between the two peaks for the nondegraded material as in model (i). *B* = σ_*d*_(1 – *R*)*I*_0_ is the degradation cross section multiplied by the radiation intensity coming into the material. μ_*s*_ is the mean macroscopic scattering cross section for the material in the diffusion approximation, and μ_*a*,0_ is the UV–vis mean absorption coefficient for the completely-degraded material. The fit results are shown in Table 4.

**Table 4:** Summary of the Fitting Parameters Using the Strongly-Degraded ABS Model

*C* (−)	*B* (1/h)	μ_*s*_ (1/μm)	μ_0,*a*_ (1/μm)	*K*_*a*_ (1/μm)
0.393	4.09 × 10^–2^	4.45 × 10^–8^	8.42 × 10^–9^	4.11 × 10^–2^

Figure 7 shows the plots of the fitted models with respect to the experimental trend. One can note that hypothesis (i), shown in Figure 7a, fails to describe the regions where the studied ABS-based samples are heavily degraded, whereas under the hypothesis of strongly-degraded material (ii), the model matches the experimental data within the measure uncertainty, as shown in Figure 7b. The model in the simplified hypothesis (i.e., considering that the absorption coefficient is unaffected by degradation) can still catch the position of steep growth front related to the penetration depth even for the most degraded sample (S6). In addition, it can also describe the early stage of degradation with comparable accuracy with respect to the strongly-degraded case, being a potential tool for investigation of materials whose degradation is still not totally developed, as is the case in many heritage-science-relevant scenarios. Moreover, the model in the strong degradation hypothesis predicts the UV–vis absorption in the effective spectral range for degradation to be almost completely dependent on the degradation state of the material. In fact, the μ_0,*a*_ parameter retrieved by the fitting procedure is negligible with respect to the *K*_0,*a*_ parameter, which relates to a deeper penetration of the oxidizing radiation while the material becomes more degraded. The decrease of the absorption coefficient seems to contradict the experimental data of the absorption coefficient of degraded pure ABS, which is shown to increase in the range of interest upon degradation.^28^ However, the absorption coefficient appearing in our model describes not a distinct optical response of the polymer molecules but rather a bulk property of the composite material (i.e., the material composed of ABS, ABS opacifiers, and pigments). Moreover, while the overall absorption coefficient in the whole considered UV–vis spectrum (λ = 320–800 nm) could still increase during the degradation process, the light propagation coefficients inferred by the fitting procedure must be intended as those related to the spectral components that are most effective in the material degradation, i.e., the mostly energetic photons.

**Figure 7:**
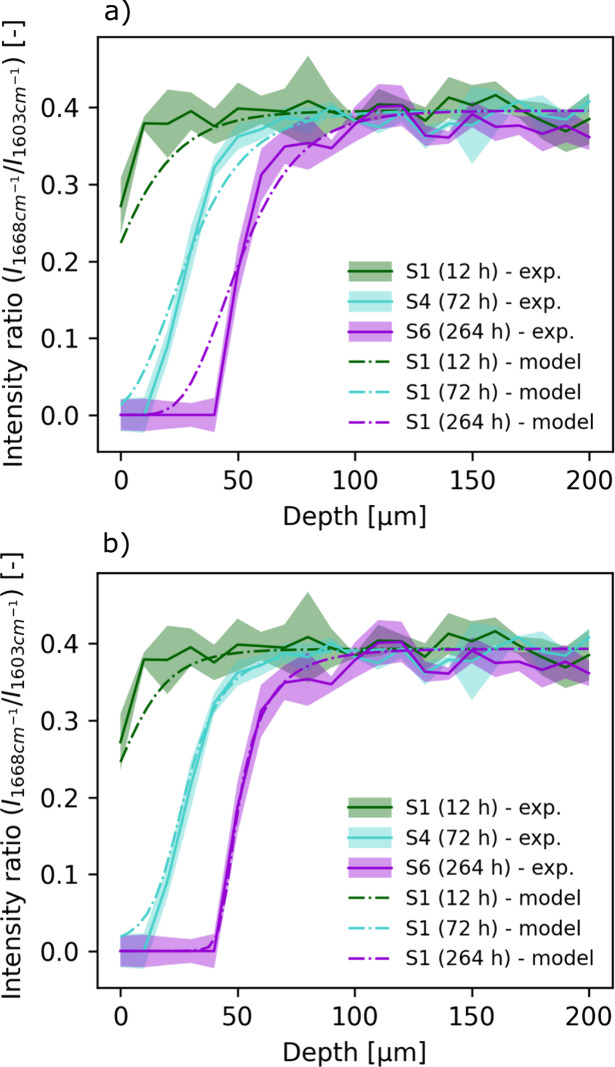
Ratio plots of the cross sections with the results from the fitted models superimposed. a) The results of the model approximated for not-strongly-degraded ABS and b) the results of the strongly-degraded ABS model.

The prediction of the strongly-degraded ABS model can be compared with the experimental observations through the SDD, GD, and MDD values, and the results are shown in Table 5. Concerning the SDD, the results from the model are comparable to the values of 10.0 ± 5.0 and 40.0 ± 5.0 μm retrieved from the experimental data and shown in Table 2. Moreover, the errors with respect to the experimental data for the two predictions are consistent with the 50% and 12.5% uncertainties for the two experimental values. As in the experimental data, no SDD is observed for the S1 model prediction. Concerning the GD, the predicted values are consistent with what were experimentally observed on S4 and S6; a difference of approximately 10 μm is instead observed between the experimental and the predicted values for S1. This higher discrepancy could be attributed to the higher uncertainty in the experimental measurements near the sample surface. Concerning the MDD, the model predicts maximum degradation thicknesses that are coherent with the experimental observations. The error in the determination is consistent with the uncertainty in the experimental data approximately equal to 16.7%, 7.1%, and 4.5% on S1, S4, and S6, respectively.

**Table 5:** Summary of the Degradation Depth Values and Errors Inferred from the Fitted Model

	SDD		GD		MDD	
Sample	SDD (μm)	Err (%)	GD (μm)	Err (%)	MDD (μm)	Err (%)
S1	-	-	15.3	168	33.9	13.0
S4	3.7	63.0	27.5	3.5	64.1	8.4
S6	41.5	3.8	51.0	0.3	86.7	13.3

#### Connection of the Degradation Model to Micro-SORS

Since the model presented in the previous section can reproduce the experimental observations with sufficient accuracy, it could be used by itself to estimate the degradation depth of degraded bricks knowing the irradiation time or the fluence. However, to noninvasively estimate the degradation profile through micro-SORS measurements, i.e., limit the number of needed cross sections, as would be needed for diagnostic campaigns on cultural heritage, we connected the degradation model (i.e., the solution of eq 6) to the material response to micro-SORS (i.e., full and defocusing micro-SORS). The connection of the degradation model to the micro-SORS measurements was performed based on the model and approximations shown by Castiglioni et al.^25^ In this case, contrarily to what is suggested in that work, we studied the intensity ratio between the 1668 and 1603 cm^–1^ bands instead of the ratio normalized to the in-focus value. Even though the latter choice allows us to avoid the fitting parameter due to the Raman cross-section ratios, it shows a diverging value for cases in which this ratio tends to zero and thus whenever the Raman signal of the C=C trans stretching vibration disappears in the in-focus measurement due to degradation.

Concerning full micro-SORS, we considered the Raman signal to be related to a precise portion inside the material, whose depth and volume vary with the offset between the illumination and collection point. Thus, an average of the signal from the region of interest was performed. Such an averaged signal can be computed by an integral average of *r*(*t*, *z*) in the interrogated volume. Moreover, as suggested by Castiglioni et al.,^25^ the depth investigated by a micro-SORS measurement can be related to the offset with a linear relation. Thus, we considered the integration extremes to have a linear relation with the offset. In particular, the lower and upper limits are related to the offset with different coefficients, and we impose the lower limit to be equal to 0 μm for the 0 μm offset. In this way, the offset measures are related to a mean interrogated depth which increases linearly with the offset, as suggested in the work. In addition, the volume interrogated by the measure increases with the offset, as expected by Monte Carlo simulations.^25^ A correction factor to the integral average is also applied to account for the possible differences in the autoabsorption of the Raman photons related to the CC stretching of the phenyl ring and the C=C trans vibration modes of butadiene. In fact, experimental observations showed a slightly descending trend with increasing offset of the intensity ratio in the micro-SORS series of the unaged sample (UA). This suggested a variation of this ratio with the interrogated volume not dependent on the degradation and not consistent with the cross-section data, which show instead a constant ratio for the nondegraded material even in depths inside the material. However, it can relate to a higher absorption of the photons Raman scattered by the C=C trans vibration modes of butadiene with respect to the ones scattered by benzene ring CC stretching during their path to the surface. Thus, the process is approximately modeled with an exponential trend given by the functional form *A* + (1 – *A*) exp­(−*BY*).

Under these hypotheses and considering as before that the phenyl ring is unaffected by the UV exposure, the intensity ratio between the C=C trans vibration modes of butadiene and the ring CC stretching (*R*(*t*, *Y*)) measured with the full micro-SORS can be represented by the following integral:

7 $$R\left(t,Y\right)=\frac{\int_{FY}^{C+kY}\sigma_{Raman,\mathrm{C=C}}n_{\mathrm{C=C}}\left(t,z\right)\;dz}{\int_{FY}^{C+kY}\sigma_{Raman,ring}n_{ring}\;dz}\left(A+\left(1-A\right)\;\exp(-BY)\right)=\frac{\int_{FY}^{C+KY}r\left(t,z\right)\;dz}{C+\left(k-F\right)Y}\left(A+\left(1-A\right)\;\exp(-BY)\right)$$

where *C*, *K*, *F*, *A*, and *B* are constants that can be fitted from the experimental data, *Y* is the offset between the illumination and collection points, and *r*(*t*, *z*) is the intensity ratio computed through eq 6. The parameter *C* is related to the depth investigated by the in-focus measurement, *K* to the linear increase of the maximum investigated depth with the offset, and *F* to the linear increase of the minimum investigated depth with the offset. *A* is the plateau value for *R*(*t*, *Y*) for high offsets, and *B* is the decay constant of the autoabsorption correction. The fitted parameters are presented in Table 6, and the results of the fit are shown in Figure 8a, comparing the model to the respective offset measures.

**Figure 8:**
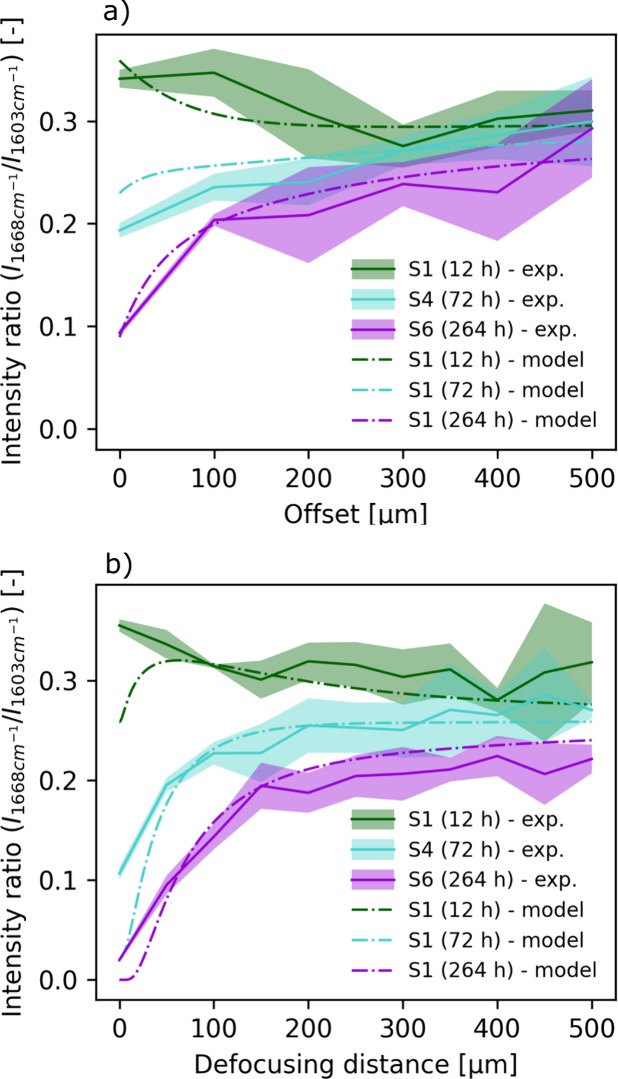
a) Ratio plot of the full micro-SORS with the results from the fitted model from eq 7 superimposed. b) Ratio plot of the defocusing micro-SORS with the results from the fitted model from eq 9 superimposed.

**Table 6:** Summary of the Fitting Parameters Using the Full Micro-SORS Model

*F* (−)	*C* (μm)	*K* (−)	*A* (−)	*B* (1/μm)
2.61 × 10^–3^	69.1	0.745	0.763	1.50 × 10^–2^

Concerning the defocusing micro-SORS, a model based on work by Castiglioni et al.^25^ was used to model the collection. In this case, the paraxial approximation for Gaussian beams was used in the place of the geometric optics approximation for describing the laser spot variation with the defocusing to avoid the laser spot reaching the 0 μm value for the in-focus measurement, thus making the integral ratio coincide to *r*(*t*, 0). With this approach, the transport kernel *G* ^*X*^(*z*) defined by Castiglioni et al.^25^ becomes

8 $$G^{X}\left(z\right)=G_{0}e^{-z/kD}=G_{0}\;\exp\left(-\frac{z}{k\frac{D_{0}}{X_{R}}\sqrt{X_{R}^{2}+X^{2}}}\right)=G_{0}\;\exp\left(-\frac{z}{\frac{\gamma }{X_{R}}\sqrt{X_{R}^{2}+X^{2}}}\right)$$

where *k* is a constant related to the linear relation between the laser spot diameter and the material depth, *D*_0_ is the in-focus spot diameter, *G*_0_ is a constant that is supposed to be equal for the photon scattered by the ring and the C=C trans vibration modes of PB in the material, *X*_*R*_ is the Rayleigh length for the laser beam, *X* is the defocusing distance, and γ = *k* *D*_0_. Under these hypotheses, considering the same autoabsorption correction factor defined for the full micro-SORS and considering that the benzene ring is unaffected by the UV exposure, the intensity ratio between the C=C trans vibration modes of butadiene and the phenyl ring CC stretching (*R*(*t*, *X*)) measured with the defocusing micro-SORS can be represented by the following integral:

9 $$R\left(t,X\right)=\frac{\int_{0}^{+\infty }\sigma_{Raman,\mathrm{C=C}}n_{\mathrm{C=C}}\left(t,z\right)G_{0}\;dz}{\int_{0}^{+\infty }\sigma_{Raman,ring}n_{ring}\;\exp\left(-\frac{z}{\frac{\gamma }{X_{R}}\sqrt{X_{R}^{2}+X^{2}}}\right)\;dz}\times \left(A+\left(1-A\right)\;\exp(-BX)\right)=\frac{\int_{0}^{+\infty }r\left(t,z\right)}{\frac{\gamma }{X_{R}}\sqrt{X_{R}^{2}+X^{2}}}\left(A+\left(1-A\right)\;\exp(-BX)\right)$$

where *A* and *B* are two constants fitted from the experimental data to account for a correction factor for defocusing measurements, with the same considerations as for full micro-SORS measurements. *r*(*t*, *z*) is the intensity ratio computed through eq 6. The fitted parameters are presented in Table 7. The results are shown in Figure 8b, comparing the fitted model to the respective defocusing measures. A mismatch from the experimental data is observed for low defocusing distances. This behavior might derive from the inability of the paraxial approximation to accurately describe tightly focused laser profiles such as the one considered, or a non-Gaussian laser spot (e.g., top-hat or super-Gaussian profiles for the focal spot whose paraxial approximation provides no simple analytical expression).

**Table 7:** Summary of the Fitting Parameters Using the Defocusing Micro-SORS Model

*X*_*R*_ (μm)	γ (μm)	*A* (−)	*B* (1/μm)
2.64	1.82	0.705	6.18 × 10^–3^

#### Proof-of-Principle of Noninvasive Protocol Application

Once the degradation model and micro-SORS collection parameters were defined, we were able to connect the micro-SORS measurements to quantitative information on the subsurface state. Thus, micro-SORS measurements could be used to estimate the degradation spatial distribution, considering the Raman intensity ratio of the 1668 and 1603 cm^–1^ peaks. To test this capability, we took as proof-of-principle the micro-SORS measurements of the S5 sample, whose cross-section data are presented in Table 2. Since both the micro-SORS and the degradation model parameters were known by the previous fitting procedures, here, the only free parameter to be considered is the irradiation time, i.e., the UV–vis fluence. Thanks to eq 6, the irradiation time is uniquely related to a degradation profile, and a minimization of the error based on DE was used on the full and defocusing micro-SORS data to evaluate the corresponding degradation profile. The results of the fit procedures are shown in Figure 9.

**Figure 9:**
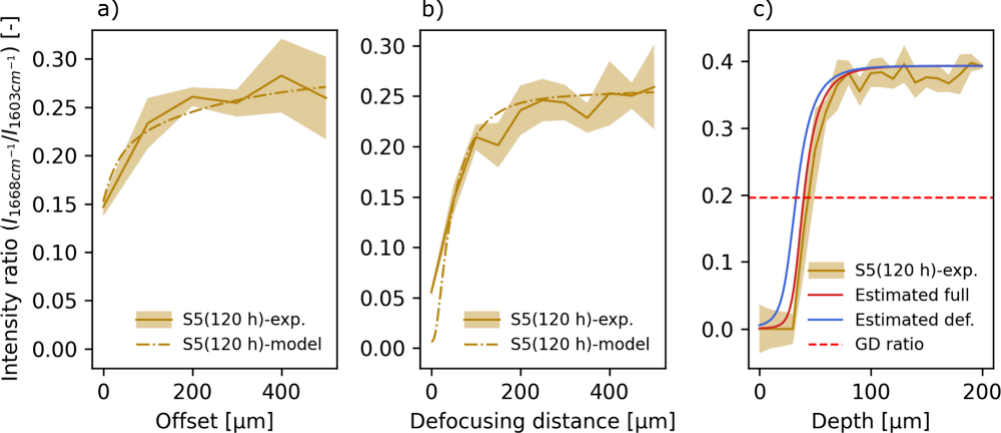
a) and b) The fitting of the models respectively from eqs 7 and 9 on experimental data from full and defocusing micro-SORS on the S5 sample. c) The respective solutions of eq 6 considering the time estimated by full and defocusing micro-SORS, compared with the cross-section data.

The fitting procedure estimates a degradation time of 145 h for the full micro-SORS and 97 h for the defocusing micro-SORS, consistent with the experimental value of 120 h. The values of SDD, GD, and MDD are summarized in Table 8. The higher errors in the defocusing modality (up to 50% for the SDD value, the most affected by errors) can be easily connected to the incomplete separation between the contributions in the Raman signal from the surface and the deeper region. Conversely, the full micro-SORS modality shows a higher separation of these components, allowing for more accurate predictions by the model. In addition, the discrepancy between the model and the low defocusing distances could produce an increased error in the fitting procedure, biasing the degradation time toward lower values. Nevertheless, the applied protocol allowed us to noninvasively estimate the experimental data within an acceptable error using both modalities, making both of them interesting for applications to design object conservations. In fact, while the full modality enables a more precise estimation of the degradation depth that might be needed, for example, when planning conservation treatments, the relative simplicity of the defocusing modality setup, which can be performed with conventional instrumentation, and the reduction of the measurement time due to the increased collected light with respect to offset measurements might be useful to monitor the degradation state over time.

**Table 8:** Summary of the Degradation Depth Values and Errors Inferred from Micro-SORS on S5

	SDD		GD		MDD	
Instrument	SDD (μm)	Err (%)	GD (μm)	Err (%)	MDD (μm)	Err (%)
Full micro-SORS	25.5	15	39.8	9.5	77.3	3.4
Defocusing micro-SORS	13.7	51	32.8	25.5	71.6	10.5

## Conclusions

This study demonstrates the potential of micro-SORS combined with simplified physical modeling as a noninvasive analytical tool for quantitatively studying acrylonitrile–​butadiene–styrene (ABS)-based materials subjected to artificial photoaging. The proposed approach suffers from some limitations including the strong sensitivity of micro-SORS to the optical properties of the analyzed material, such as scattering and absorption coefficients, which may vary with ABS formulation, color, or opacifier content. Thus, the proposed calibration cannot be generalized to all ABS-based objects. Moreover, the proposed degradation model suffers from limitations in its application due to the simplifications made in the description of the photo-oxidation mechanism.

Despite these constraints, the method holds significant promise for application to design objects produced in multiple replicas, where a limited number of sacrificial samples can be used to calibrate the protocol. This calibration enables the noninvasive estimation of the degradation depth to sets of similar objects, commonly found in museums, where collections of serial items have been gathered with each individual piece experiencing different conservation histories. In addition, the method can be exploited for long-term monitoring by reanalyzing the same objects over time with no intervention. Nevertheless, the measurement of the scattering and absorption coefficients through experiments, together with a more complex modeling of the degradation—explicitly considering the effects of the oxygen diffusion—and of the micro-SORS measurements, e.g., via Monte Carlo simulations, could allow for a completely noninvasive approach to the study of ABS plastic degradation without the need of cross sections. Moreover, the creation of a database containing the optical and degradation parameters for standard ABS formulations could avoid the use of sacrificial samples for many objects even within this simplified approach. In this sense, our work represents a first step toward the noninvasive study of ABS object degradation based on a physical modeling of the process.

The study identifies potential instrumentation enhancements, specifically the adoption of higher magnification objectives to increase spatial resolution in both the benchtop prototype for full micro-SORS measurements and the portable prototype. Moreover, these results suggest that, despite the lower spatial resolution in focus and at low offsets, the full micro-SORS modality remains the most appropriate to be coupled to the model; in fact, its higher ability to discern the signal from different regions of the material allows for a more accurate estimation of the degradation profile. Overall, the developed protocol represents a significant advancement in nondestructive diagnostics for ABS degradation, laying the groundwork for a broader adoption of micro-SORS in the monitoring and preservation of modern polymeric materials in cultural heritage.

## Supplementary Material



## Notes

Additional experimental details, materials, and methods, including ratio plots obtained using the intensities of Raman bands assigned to the C=C stretching vibrations of the cis and trans configuration and to the vinyl group of polybutadiene units; description of the automatized fitting procedure for Raman spectra; efficiency tests for the fiber bundle in the portable prototype; and comparison of raw spectra of the same sample collected by the benchtop and portable instruments (PDF)

## #

Historic England, Portsmouth PO4 9LD, United Kingdom
