# Supplementary material for: Micro-SORS-Based Protocol for the Noninvasive Depth-Resolved Study of Degraded ABS in Cultural Heritage
Source: Anal Chem. 2026 Jul 14;98(29):21219–30. doi: 10.1021/acs.analchem.6c00327 (PMC13425560; doi:10.1021/acs.analchem.6c00327)
Supplement: Supplementary file 1 [file ac6c00327_si_001.pdf]

Supplementary materials

Supporting information: "Micro-SORS based protocol for the non-invasive depth-resolved study of degraded ABS in cultural heritage"

Kevin Ambrogioni[a*], Chiara Castiglioni[b], Francesca Rosi[c], Claudia Conti[d], Matteo Passoni[a], Pavel Matousek[e], Irene Bargagli[c,†], Alessandra Botteon[d]

[a] Politecnico di Milano, Department of Energy, via Lambruschini 6, 20156, Milano, Italy

[b] Politecnico di Milano, Department of Chemistry, Materials and Chemical Engineering Giulio Natta, piazza Leonardo da Vinci 32, 20131 Milano, Italy.

[c] National Research Council, Istituto di Science e Tecnologie Chimiche "G. Natta" (CNR-SCITEC), Via Elce di Sotto 8, 016280 Perugia, Italy

[d] National Research Council, Institute of Heritage Science, via Roberto Cozzi 53, 20125 Milano, Italy

[e] Central Laser Facility, Research Complex at Harwell, STFC Rutherford Appleton Laboratory, UKRI, Oxford OX11 0QX, U.K.

[†] current address: Historic England, Portsmouth PO4 9LD, U.K.

* Corresponding author: kevin.ambrogioni@polimi.it

Table of contents

Supplementary text
Figure S1
Figure S2
Figure S3
Figure S4

Supplementary materials

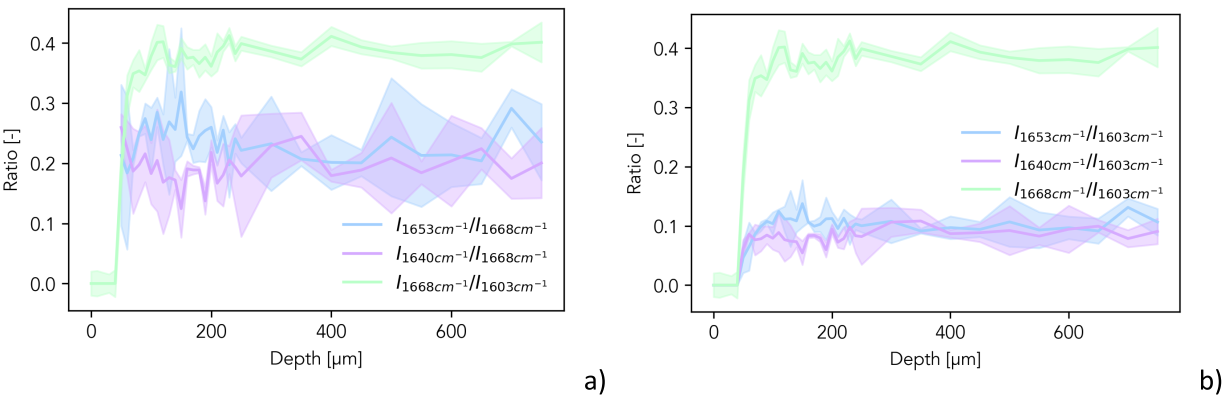

Figure S1. *a) Ratio plot obtained using the intensities of Raman bands assigned to the C=C stretching vibrations of the cis (1653 cm^{-1}) configuration of 1,4 polybutadiene units and to the vinyl group (1640 cm^{-1}) of the 1,2 units, and Raman bands assigned to the C=C stretching vibrations of the trans (1668 cm^{-1}) configuration of 1,4 polybutadiene units. b) Ratio plot obtained using the intensities of Raman bands assigned to the C=C stretching vibrations of the trans (1668 cm^{-1}) and cis (1653 cm^{-1}) configuration of 1,4 polybutadiene units and to the vinyl group (1640 cm^{-1}) of the 1,2 units, and the intensity of the Raman band 1603 cm^{-1} assigned to the aromatic rings belonging to the polystyrene segments.*

Automatised fitting procedure for Raman spectra

The code proceeds in two main steps:

1. The baseline removal.
2. The fitting of the region of interest.

The baseline correction is based on the statistics-sensitive non-linear iterative peak-clipping (SNIP) algorithm. To avoid the typical errors arising with this baseline correction method when high-frequency noise is present[1], the SNIP algorithm is applied on a smoothed spectrum. The smoothing procedure is based on an adaptive Gaussian filter, developed to smooth the typical etaloning shapes appearing at high wavenumbers.

Following this, a fit is performed on the baseline corrected spectrum. In this step, the non-filtered spectrum is used. The peak is modelled with a Voigt profile. The fitting procedure consists of two steps. The first step uses the differential evolution (DE) algorithm to fit the bands of interest, i.e., an evolutionary algorithm. This algorithm allows the fitting parameters to vary in a bounded region and iteratively chooses the best options among a user defined number of possible solutions. By iterating up to satisfying a tolerance criterion, the algorithm minimises the residuals and finds the candidate solution. Differently from the gradient descendent methods, the DE algorithm is less sensitive to local minimum of the objective function[2], thus, being more effective in automatised analyses. The second step of the fit is based on the trust-region reflective (TRF) algorithm, a gradient descendent algorithm. We bind the solution for the TRF in the neighbourhood of the result from DE, using the latter as the guess parameter for starting the gradient descendent method. The TRF algorithm is allowed to fit all the peak parameters except for its position and is used to estimate the errors of the fitted parameters. The results from the fit are used to compute the intensity ratio of the peaks (Figure S2b).

Supplementary materials

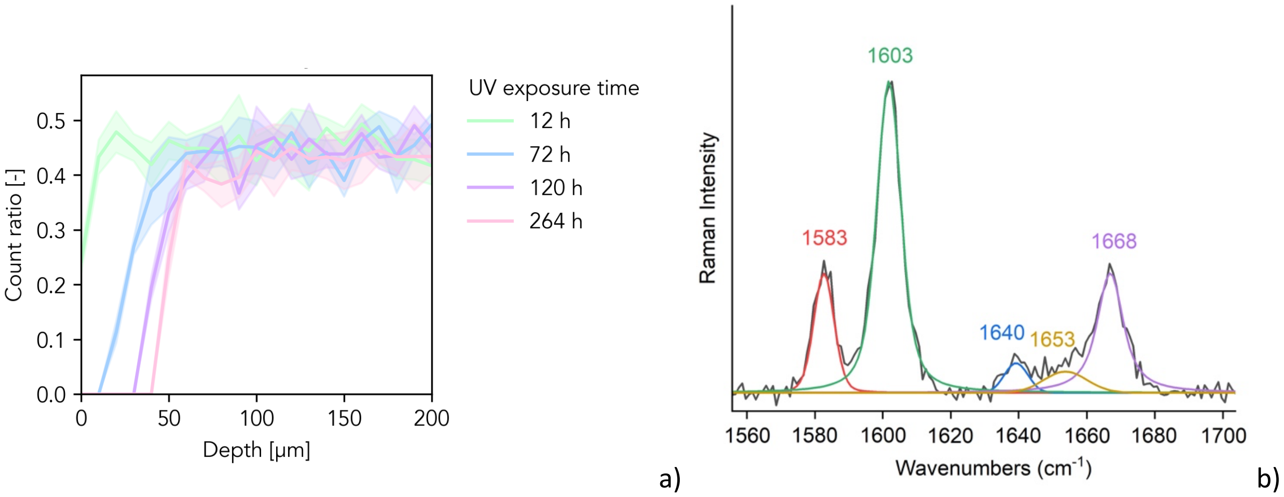

Figure S2. *a) Ratio of the Raman band integral between the bands relative to the polybutadiene double bonds and the polystyrene phenyl ring. b) Raman spectrum collected on the cross-section of S6 (264h) fitted with the procedure described in section 2.4.*

Fibers bundle efficiency test

When concerning the portable prototype, the presence of the fibre bundle in the collection might generate some distortion on the calculation of such Raman intensity ratios if the photon transport efficiency varies across the different fibres. To assess whether a correction factor is needed for these ratio trends, we calculated the photon collection efficiency of the fibre bundle in the portable prototype. We used a diffused light source, produced by broadband light source (100-W bulb with a tungsten filament). We placed the light bulb in front of the microscope objective used for collection, and we acquired the spectra for 1 s. The acquisition was repeated 2 times.

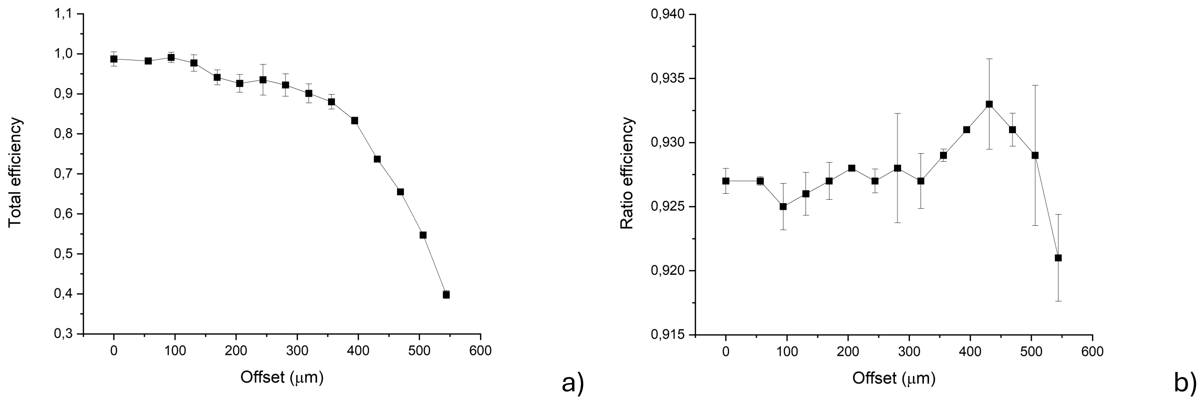

Figure S3. *a) total and b) relative efficiency of the fibre bundle. The markers represent the average efficiency value for the two takes, the error-bars represent the standard deviation.*

The total efficiency of the fibre bundle in each acquisition was estimated by integrating the total signal from the diffuse spectrum of the light bulb and dividing it by a reference value, i.e. the integrated counts from the fibres at 0.0-μm offset. The plot in Figure S3a shows the average efficiency value between the two takes and the standard deviation. The collection was performed pairing the fibres with respect to the centre, which is considered as the in-focus point. The total efficiency slowly varies across the

offsets; in fact, only the four more external offsets (respectively 431.25 μm, 468.75 μm, 506.25 μm and 543.75 μm) seem to heavily deviate from unity. Instead, concerning the relative intensity between the regions of interest for the following analysis (Figure S3b), we evaluated the relative efficiency as the ratio of the total signal in the region between 1550 cm^{-1} and 1620 cm^{-1} over the total signal between 1620 cm^{-1} and 1690 cm^{-1}. Again, we averaged this value for the two acquisitions and computed the standard deviation. The relative efficiency can be considered constant up to an offset of 431.25 μm. Thus, the evaluation of the ratios between the two bands of interest should not be affected by differences in the efficiency when keeping a maximum offset of 431.25 μm.

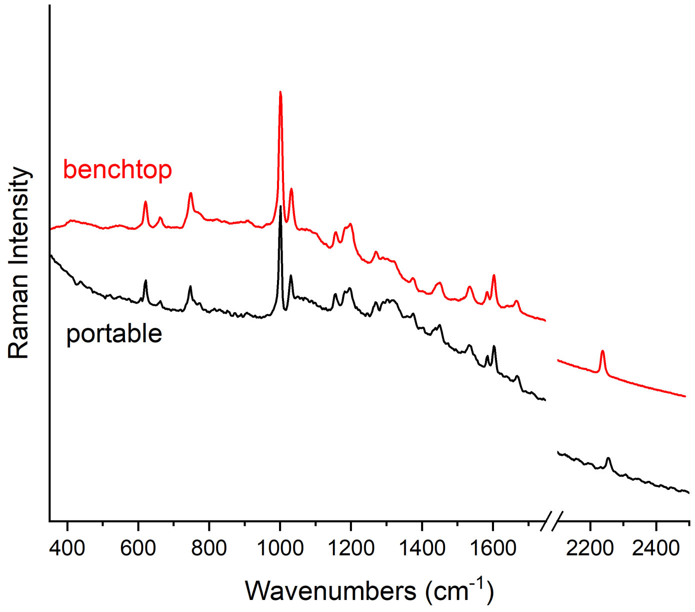

Figure S4. *Comparison between Raman spectra collected at the surface of UA Lego brick with the benchtop and portable prototypes.*

[1] Mostafapour S., Dörfer T., Heinke R., Rösch P., Popp J., Bocklitz T. (2023). Investigating the effect of different pre-treatment methods on Raman spectra recorded with different excitation wavelengths. *Spectrochimica Acta Part A: Molecular and Biomolecular Spectroscopy*, 302(123100). https://doi.org/10.1016/j.saa.2023.123100.

[2] Salomon R. (1998) Evolutionary algorithms and gradient search: similarities and differences. *IEEE Transaction on Evolutionary Computation,* 2(2). https://doi.org/10.1109/4235.728207
